# Book Reviews

**Published:** 1877-01

**Authors:** 


### Book Reviews.

[NOTE.—All works reviewed in the pages of the CHICAGO MEDICAL JOURNAL AND EXAMINER may be found in the extensive stock of W. B. KEEN, COOKE & Co., whose Catalogue of Medical Books will be sent to any address upon request.]

GUY'S HOSPITAL REPORTS, edited by *H. G. House, M.S.*, and *Frederick Taylor, M.D.* Third series. Vol. XXXI. 8vo. pp. 469. London: J. & A. Churchill. 1876.

We have read this volume with great pleasure. It is one of the most valuable of the recent contributions to medical literature. The contents are particularly rich in original investigations.

The first article is entitled "Cases Illustrating the Diuretic

Action of the Resin of Copaiba," by Frederick Taylor, M.D. The resin of copaiba is the residue of the distillation of oleum copaibæ, is described as of a dark green color, brittle, resinous in consistence, and almost tasteless. In preparing it for use the resin is softened with one-fourth of its bulk of rectified spirits; three ounces of this mixture are rubbed with four ounces of compound tragacanth powder, and mixed with four pints of water. One ounce of this mixture contains 12 or 13 grains of the resin, and was given three times daily. This preparation was administered in more than sixty cases, of hepatic ascites, simple peritoneal effusion, cardiac dropsy, anasarca, ascites secondary to emphysema and bronchitis, pleuritic effusion and renal dropsy. In favorable cases the quantity of urine was quickly increased. For example, in case 5, the urine passed the day before the drug was first administered, measured 18 ounces, and on the following day, 76 ounces. In case 6, the urine was increased from 60 ounces to 122 ounces on the following day. The diuresis thus rapidly produced, subsided as rapidly when the drug was withdrawn.

There are thirteen cases recorded in which the drug was used in ascites from disease of the liver. Commenting on these cases, our author says: "the results of the first ten cases appear to contradict very decidedly the assertion of Niemeyer, that diuretics are as useless as they are irrational in the treatment of ascites." The group representing its use in cardiac dropsy, is interesting because of the comparisions instituted between it and digitalis, with apparent advantage in favor of copaiba. As an example, in case 17, during the first nine days with the use of digitalis, there was a daily average of 23 ounces urine, but on the addition of copaiba resin, it increased rapidly to 90, 100 and 125 ounces. In case 16 the urine increased to 110 and 120 ounces under the combined use of digitalis and resin copaiba, but on the withdrawal of the latter fell to 40, 20 and 16 ounces, and rose to 80 ounces when it was again employed.

In renal dropsy the drug had no appreciable diuretic effect in three cases of tubular nephritis, but seemed to have considerable influence in one case of lardaceous disease and in one of granular disease. In none of the cases did positive harm follow its use.

The second article is entitled "Considerations on the Cures in Insanity," by George H. Savage, M.D., and is quite an exhaustive dissertation on prognosis, the result of ten years' experience in the Bethlem Royal Hospital, by a very careful and accurate observer. The limits of this review are not sufficient to enable us to give an outline that will do it justice. There is one important fact brought out in connection with treatment of the insane, that we think of considerable value, it is the good result that sometimes follows the transfer of the patient from one asylum to another. A personal experience with these unfortunates has given us several cases in which recovery quickly followed such transfer. Our author remarks, "that the prognosis is not absolutelly bad when patients have already been discharged uncured and incurable from an asylum. I have many times been annoyed to hear of patients who, after all our care and trouble, have not been benefitted, and yet have recovered on removal to rougher quarters and harder fare." At the Metropolitan Asylum, at Leavesden, only presumably incurable patients are sent, yet some are every year discharged cured.

"One is not only justified but encouraged to send to other asylums those cases that we have failed in curing at the end of a year." The records of our American Asylum for Incurables, at Ovid, N. Y., give the same results as those observed at the English institutions.

The next article is "On Fractures of the Thigh," by J. Cooper Forster, Esq. In it is considered, with great ability, and with an experience of thirty years' observation, in an excellent clinical field, the treatment most certain to prevent shortening. He first discusses the mechanism and results of treatment with the double inclined plane and the long straight splint, and then considers the splint devised by Dr. Smith, of Baltimore, as modified by Dr. Hodgen, of St. Louis, and regards this latter as the best appliance before the profession, fulfilling as it does, in a scientific manner, the indications of muscular relaxation, rest and extension. Our author has treated, with this splint, 17 cases, with an average shortening of only one-third of an inch. In contrast with this he gives the record of 47 cases treated by various other plans, in which the shortening averaged three-quarters of an inch. He considers the Hodgen splint as the one most comfortable to the patient, admitting of the greatest freedom of body movement with least disturbance of fracture, and greatest degree of cleanliness. He enumerates, as its disadvantages, the thought and dexterity its application requires, the difficulty of obtaining the material for fitting it up, the liability to excoriation of the leg from the extension straps, and the care necessary to prevent eversion of the lower fragment with the whole of the foot.

A personal experience of the reviewer with the splint, in three cases of gunshot fracture of the femur, leads him to fully endorse all that is said in its favor.

The next article "On Meningeal Hæmorrhage," by James F. Goodhart, M.D., is an admirable resumé of 49 cases. He lays special stress on the fact that these hæmorrhages are probably often present without being suspected; that they occur from apparently trivial accidents, and that if care is not exercised, cases which might have perfectly recovered pass on into a state of permanent degeneration of the grey matter of the brain, and even into a state of chronic inflammation of the brain and its membranes, these leading ultimately to confirmed epilepsy, to insanity, and even to death.

The next paper is "On the Causes of Preventable Blindness," by C. Higgins, Esq. The object is to call attention to certain affections of the eyeball and its appendages which, if proper precaution be taken, may leave no bad results; but which, if neglected or improperly treated, will lead to more or less impairment of function, and admirably has he stated his observations.

He details his experience in granular ophthalmia, purulent ophthalmia and glaucoma, and makes valuable suggestions as to prevention and treatment. He justly condemns strong caustic applications to the conjunctiva, and gives, as the usual treatment for out patients, the following: "In the more recent cases the palpebral conjunctiva is twice a week touched lightly all over with mitigated nitrate of silver stick (one part of nitrate of silver to three of nitrate of potash); after the application the conjunctiva is washed with salt and water. In the more chronic cases the green stone (lapis divinus) is used instead of the nitrate of silver. In most cases sulphate of copper drops (cupri sulph., gr. ij., aqua, ℥j), are ordered to be dropped into the eyes three times a day or oftener. If there is much intolerance of light, or symptoms of iritis exist, gr. ½ to j of sulphate of atropine is added to each ounce of the drops. If there be copious purulent discharge, alum lotion (gr. 10 to ℥j) is ordered in lieu of the drops. If extensive ulceration of the cornea exists, the eye is to be kept bandaged with lint soaked in belladonna or poppies. The granulations are neglected until the more severe symptoms have subsided."

The treatment he prescribes in ophthalmia neonatorum is the use of alum lotion (gr. ij to ℥j) every hour or half hour, according to amount of discharge from 8 A. M. to 8 P. M., and during the night when the child is awake. In cases where deep ulceration or perforation has taken place, he orders the eye to be bandaged with lint soaked in belladonna lotion, the bandage to be frequently removed in order to apply the alum lotion.

In mild cases of purulent ophthalmia he recommends the use of alum or other astringent lotions frequently applied; in severe cases a thorough application of solid nitrate of silver to the palpebral and ocular surface of the conjunctiva, followed by a washing with salt and water. The eye is then to be lightly covered with a piece of lint and kept constantly bathed with alum lotion (gr. x to ℥j), some simple ointment to be applied to lids and cheek; quinine, iron, a liberal diet with stimulant and opium at night are also prescribed.

The next cause of preventable blindness he treats of, is *undetected glaucoma.* He enumerates several cases in which the disease was mistaken for senile cataract, and appropriate treatment thereby delayed until too late for successful result. This should warn us against a hasty diagnosis of cataract from unaided eye appearances, and cause every suspected case to be subjected to a careful ophthalmoscopic examination. The only successful treatment is operative, and this of no value unless early applied.

The contributions to dental pathology by S. James A. Salter, M.B., F.R.S., and "A Description of the Appearances of the Human Eye in Health and Disease, as seen by the Ophthalmoscope, ninth series, Retinitis Pigmentosa," by C. Bader, Esq., are both very entertaining papers.

The next article is on the "Use and Administration of Sedatives," by Paul Henry Stokoe, B.A., M.D., and is a truly valuable paper. In treating children in their first septenate, he rejects opium almost entirely for internal use. In regard to opium in second childhood, he considers it judicious to combine chloral and chloroform with it; opium being in excess when pain, chloral when restlessness, and chloroform when cramp predominates; and furthermore recommends the addition of m. x to xx of the tincture of cannabis Indica, when dealing with a heart enfeebled by advanced age or exhausting illness. In this connection we feel it our duty to give a word of warning in the use of chloral. We are aware that many practitioners regard it as harmless and freely prescribe it in doses that we consider dangerous. We have had a somewhat extensive experience in its use, especially in the insomnia of the insane, we have seen dangerous symptoms result from a 20 grain dose, and have been told of a death that resulted from one dose of 20 grains administered to a healthy woman to allay the pain of a tooth extraction. We have also known 40 grains result in poisoning, from which the patient was saved only by prompt interference. These are exceptional cases it is true, but such is our experience, and we would hesitate ever to administer to an adult, a dose exceeding 10 grains at one time.

The next article is entitled the "Fifth Report of the Guy's Hospital Lying-in Charity," collated by A. N. Galabin, M.D. It extends over a period of 12 years, and embraces 23,591 deliveries. The total number of children born is 23,811; of these 95.92 per cent. were born alive, the sexes were in the proportion of 100 males to 88 females; 97.75 per cent. of the living children and 64.4 per cent. of still born children were delivered under a vertex presentation. There were 220 twin cases, or 1 in 107 of the whole number of women delivered. Only one cases of triplets occurred. The proportion of forceps cases was 1 in 197; out of the 121 forceps cases, five of the mothers died, one from hæmorrhage, one undelivered and three from septicæmia. The long curved forceps of Dr. Barnes were almost invariably used, even when the head was arrested at or near the outlet. These records seem to show that the application of the forceps is not necessary in as large number of cases as some of our own obstetricians would have us believe. Craniotomy was performed in 18 cases, or 1 in 1310. In fifteen the operation was resorted to for want of due relation between the head and passages, in one for compound presentation of head and foot, in one for carcinoma of the cervix uteri, and in one for the delivery of a hydrocephalic head — there were six deaths. There were seven cases of rupture of uterus and vagina — all died. As might have been expected from the enfeebled and badly nourished character of the patients, post partum hæmorrhage was of frequent occurrence, and caused death in eleven cases. Transfusion was tried in five cases, but in all was unsuccessful. A solution of perchloride of iron was injected into the uterus as a last resort in twelve cases, and in all instances stopped the bleeding — one-half of the cases died, but the injection does not appear to have itself contributed to the fatal issue. Forty-one cases of placenta prævia occurred. Two of the cases are remarkable in that the os was partially covered by the placenta and no hæmorrhage had taken place. Version was performed in 24 out of the 41 cases. Six of the mothers died, 10 of the children were delivered living and 31 were still born. The placenta was adhered and required the introduction of the hand for its removal in 75 cases, with five deaths, three from post partum hæmorrhage and two from puerperal peritonitis. Twenty-eight cases of eclampsia are recorded; this does not include, however, all the slight cases that recovered. There were seven deaths among mothers. In all cases in which urine was examined albumen was found, except in one instance. In this internal hæmorrhage occurred after delivery, and epileptiform convulsions followed; the pulse being dicrotic and compressible, very unlike that generally found in eclampsia. It is moreover interesting to note that in all the cases that have occurred during the last 40 years, in which urine was examined, albumen was found in every case but two, one, the case above mentioned, and the other a case of arachnitis, as verified by autopsy.

Our author tells us that since 1868 no patient has been bled, but reliance has been placed on the administration of chloroform, often for many hours consecutively, even when the element of coma predominated over that of convulsion. Throwing out two fatal cases in which there was no opportunity for the proper administration of the remedy and one case that was an old epileptic, and the mortality under chloroform treatment is only 9 per cent. against 30 per cent. in which bleeding was used. There were 34 fatal cases out 36 cases of puerperal peritonitis. The usual treatment was quinine, opium and intra-uterine injections, with the occasional use of sulpho-carbolate of soda.

The mortality from all causes was 1 in 223; a result quite creditable when we consider that the patients are drawn from one of the worst neighborhoods in London, so far as extreme destitution and hygienic conditions are concerned. Rather less than half the deaths were due to septicæmia, and nearly a quarter to hæmorrhage.

The next article is entitled "The Treatment of Ulcers by the Local Application of a Weak Continuous Electric Current," by C. H. Golding Bird, B.A., M.D., and is a record of six cases treated successfully by this method.

The next article is entitled "A Case of Fracture of the Skull, Followed by a Collection of Cerebro-Spinal Fluid Beneath the Scalp." Recovery. By R. Clement Lucas, B.S. The tumor occupied the left temporal fossa, was aspirated and two ounces of cerebro-spinal fluid removed. The child has not spoken since accident. The case would probably have done better if the fluid had been left where nature put it.

The paper entitled "Remarks on some of the Paroxysmal Neuroses," by C. Hilton Fagge, M.D., is next in order. In it is discussed those functional diseases of the nervous centers that occur paroxysmally at periods more or less regular, "nerve storms," and includes migraine, vertigo, epilepsy, paroxysmal insanity, and tetany. He finds that they are essentially innate and hereditary, as are many of the neuroses; that in the same patient they are often transformed one into another in the course of time; that in the most of them there is, in each attack, a regular succession of phenomena, dependant, doubtless, upon a gradual extension of the morbid change from one part to another of the nervous centers, rather than modifications in the blood supply; that the attacks gradually culminate to a certain pitch of intensity and then subside; that a *slight* attack is followed at an unusually short period by another, and *vice versa;* and that the seizures are often traceable to causes which are very similar.

Space will not permit us to consider all the causes, but there are three that are well brought out in the paper that are frequently overlooked in practice: these are, derangement of the visual apparatus, defection of teeth, and the presence of tænia. The author offers nothing new on treatment.

The next paper is "On the Recognition of Sugar in Healthy Urine," by F. W. Pavy, M.D., F.R.S. It is a very valuable article, successfully answering in the affirmative the question: Does sugar exist in healthy urine? The writer describes a method of manipulation, in the test by fermentation, much superior to any that we are familiar with. Speaking of the pathological importance of the recognition of sugar as a constituent of healthy urine, he remarks that "it enables us to reconcile ourselves to the instances in which sugar is incidentally met with to a moderate extent in the urine without being associated with any clinical significance." He narrates three cases in which respectively 3.42, 2.55 and 4.44 grains of sugar to the ounce of urine were found, without any constitutional symptoms of diabetes, and which disappeared in a few days without any diatetic or other treatment for diabetes being employed.

This review, notwithstanding its length, but imperfectly represents the value of the report, and we, in conclusion, commend it to the profession as a book worth careful study.

D. R. B.

ANNUAL REPORT OF THE SURGEON GENERAL U. S. A. 1876.

In this brief report Dr. J. K. Barnes, Surgeon General U. S. A., informs us that the U. S. army showed an average mean strength of 21,681 white and 2,002 colored troops. Among the white soldiers 32,495 cases, or 1,499 per thousand, were taken on the sick report for diseases, and 5,066, or 233 per thousand, were reported for wounds and injuries of all kinds. The total number of deaths reported from all causes was 518, or 24 per thousand. These figures include 267 officers and men killed in the Indian war.

Among the colored troops the total number of cases of disease was 2,941, or 1,469 per thousand, and the number of wounds and other injuries was 521, or 260 per thousand. The number of deaths was 26, or 13 per thousand.

In all, 61 cases and 29 deaths of yellow fever occurred among the troops during the summer and fall of 1875.

During the past year 2,001 cases of wounds and injuries, and 71 cases of operations, have been added to the surgical registers, which now contain 267,700 abstracts of cases.

A TREATISE ON THE DISEASES OF THE NERVOUS SYSTEM. By *William A. Hammond, M.D., etc.* Sixth edition. 8vo., pp. 883. D. Appleton & Co., New York. 1876.

A book that has met with such a demand as to exhaust five large editions in four years, would seem to be beyond the reach of unfavorable criticism. Such is the book before us. We confess ourselves at a loss to account for such unprecedented success. Certainly we have found nothing in the volume itself that will explain it. The book contains, in our humble judgment, so much error intimately mixed with truth that it requires an adept to eliminate it, and would be in the hands of an inexperienced practitioner an unreliable guide.

The following sentence shows the laconic manner in which our author, in the beginning, disposes of his critics: "The real value of ophthalmoscopy in diseases of the nervous system is in danger of being disregarded through the sciolism of pert pretenders, who read papers and memoirs without ever having seen the optic disk to recognize it" (page 18).

Persuing the subject of ophthalmoscopy in this relation, we find, under *active cerebral congestion*, of which our author had in his private practice in five years 622 cases, that the ophthalmoscopic examination is the most important aid to diagnosis; that it "shows the arteries of the retinæ to be increased in number, diameter and tortuosity." Under partial cerebral *anœmia* from embolism, he says the ophthalmoscope should always be used; that "in the older cases we will frequently find retinal congestion." Exactly how these two revelations of the ophthalmoscope are to be reconciled with its value, as estimated by our author, we are unable to say, and are disposed to question the importance of the instrument as an aid to diagnosis, even at the risk of being called a sciolist.

If space permitted, we might say much about the unwarranted positiveness in diagnosis, pathology and treatment that pervades every chapter.

The book contains one hundred and nine illustrations, a few of which are of real scientific value; many are sensational, and some burlesque.

In conclusion, we regret that the book is not in our judgment such as we should expect to emanate from one occupying such a distinguished position in the profession as our author holds. D. R. B.

CHEMISTRY: GENERAL, MEDICAL AND PHARMACEUTICAL. By *John Attfield, Ph.D.* Seventh edition. Philadelphia: Henry C. Lea.

The enviable reputation already gained by this standard work will certainly be much increased by the many improvements incorporated in the new edition that has just appeared. A number of valuable, well executed illustrations of chemical apparatus have been added, the recent changes in the nomenclature of the United States Pharmacopœia are duly noted, and the whole work revised so as to be abreast with the many late discoveries in chemical science. At the same time the thoroughly practical character of the previous editions is still maintained—a matter that will be appreciated by busy practitioners, who, as a rule, seek from chemistries useful facts and not the discussion of unproven theories. To them the well-written chapters on toxicological testing and urinalysis will be of especial value; indeed, all that portion of the book devoted to analvtical work is excellent, and by itself alone is worth the price paid for the volume.

We are surprised, however, in a work as generally accurate as Prof. Attfield's, to find so glaring an error as that which occurs on page 481, in connection with the important subject of testing for albumen in the urine. The statement is made that the urine, previous to boiling, must be acidulated, and for this purpose nitric acid is recommended in preference to acetic. Now, it has repeatedly been pointed out by the best of chemical authorities that urine, holding in solution a not over large quantity of albumen, will frequently fail to show any trace of that substance, if before boiling it has been acidified by nitric instead of acetic acid. We know of at least one instance in which ignorance of this fact led an eminent professor of clinical medicine to declare a patient free from albuminuria, when in reality that condition existed to a well marked extent.

All things considered, however, Attfield's Chemistry, in its present revised and improved form, is certainly one of our very best medical chemistries, and we take pleasure in recommending it to the profession and to students of medicine as a very valuable book of reference. W. S. H.

THE STUDENT'S GUIDE TO DENTAL ANATOMY AND SURGERY. By *Henry Sewill*, Member of the Royal College of Surgeons, etc. Philadelphia: Lindsay & Blakiston. 1876.

This little book of two hundred pages is a model in its way. Designed for students of dentistry, and condensed into the space of a manual, it is written by a scholar and an expert in his department, and would repay a perusal by any surgeon or student of surgery. It must be invaluable to those students of dentistry who desire to be scientific men — as we are glad many of them at this day are.

Surgery of the Arteries; being the Lettsomian Lectures of the Medical Society of London, 1875. By *C. F. Maunder*, Surgeon to the London Hospital, etc.

This contribution to surgery, of 202 pages, is composed of three lectures. The time allotted for their delivery was so short as to restrict the author, in a great measure, to his own personal experience. He has been obliged, therefore, to leave untouched many points of interest pertaining to his subject. The first lecture is devoted to aneurisms; the second to wounds, hæmorrhage and the antiseptic ligature; the third to the ligation of a main artery to arrest acute traumatic inflammation. In his first lecture we find the details of cases of aortic, carotid, axillary, inguinal and popliteal aneurisms of these arteries, with their anatomical relations and the operations of ligation called for when thus affected.

The result of Robert Holmes' inquiries concerning the compression treatment will lead us, says Mr. Maunder, to be more discriminating in the selection of the means of cure to be tried first, as well as the time during which compression may be continued, it having been shown by the former that the mortality from ligature, after compression has failed, is ten per cent. greater than where the Hunterian operation has been performed at once. And after a still more extended observation of popliteal aneurism, the author repeats what he published in 1869, viz.: "The mode of treatment I advocated for the cure of popliteal aneurism and all other suitable cases, is moderate compression, alternating with relaxation, say for a fortnight, with a view, partly, if thought desirable, of promoting a more free collateral circulation in the limb; and at the expiration of this time *continuous* compression, either digital or instrumental (completely obstructing the artery), maintained under chloroform or opium, if necessary, for a period varying from six to twelve hours, or even longer, and assisted by a tourniquet on the distal side of the sac, if the first attempt did not succeed. Should a few sittings fail to effect good progress in the cure of the aneurism, the ligature must be resorted to." This lecture closes with the following conclusions:

1. "That no aneurism is to be regarded as necessarily incurable."

2. "That some cases of internal aneurism are apparently cured by absolute and prolonged rest, restricted diet, and other medical treatment."

3. "That when possible, compression, either proximal or distal, is to be employed in addition."

4. "That in all aneurisms in which treatment by ligature is known to be a fatal operation, the above rules are to be first applied."

5. "That the treatment of progressive aneurism at the root of the neck, by the distal operation, is justifiable after medical treatment has failed."

6. "That in rare instances only may an aneurism be treated by ligature before compression has been tried and has failed."

7. "That digital is to be preferred to instrumental compression."

8. "That anæsthetics and morphia are valuable aids to compression."

9. "That chloroform will probably prove to be a more effectual agent than morphia in all cases, but the more hazardous."

10. "That the value of morphia should be more thoroughly tested."

In his lecture on wounds, etc., Mr. Maunder gives due importance to the flowing of venous blood from the distal side of a wound in an artery, as an indication of the means to be employed for the arrest of secondary hæmorrhage. Experience shows that repair can be more certainly expected to occur on the proximal than on the distal side of the ligature of an artery, and to know whence the blood comes is of the utmost value to the surgeon. Should it be arterial and proximal, little else than the application of a ligature higher up the trunk will suffice to arrest the bleeding occasionally.

The prognosis will be much more favorable if the blood be of a dark color, when rest and the local application of cold and pressure will be effectual. One case is cited to prove that venous blood sometimes flows from the distal side of the wound, and one to illustrate the value of the color of the blood as an indication of the method to be employed. From the cases of hæmorrhage cited he draws various conclusions, from which we select the following:

VI. "That blood flowing from the distal side of a wound in an artery or ligature upon it, will in the lower extremity be often, in the upper extremity occasionally, venous in color."

X. "That both the axillary and the femoral arteries may be wounded, and a pulse be felt at the extremity of the limb."

XII. "That direct compression upon the bleeding point will often succeed *after* the main artery has been tied, though it failed *before;* and this fact is a justification for tying a main vessel."

Part of this lecture (second) is devoted to the use of the antiseptic ligature upon arteries in their continuity. Nineteen arteries are reported as tied antiseptically—four with the antiseptic silk ligature (silk soaked in carbolic acid solution), and fifteen with antiseptic cat-gut. In eleven of the latter, and in all of the former, the behavior of the ligature was satisfactory. The four remaining ligations with the cat-gut furnish evidence adverse to this variety of ligature. Mr. E. Watson, for popliteal aneurism, applied an antiseptic cat-gut ligature in Hunter's canal, but without arresting pulsation beyond a few seconds. Five days later a similar operation was performed upon the ext. iliac artery. A month later the patient died, after amputation. The latter vessel, when slit up, had its lining smooth and unbroken, and there was no evidence of diminution of the caliber of the vessel. No trace of the cat-gut was discovered. The opinion entertained was that the ligature did not confine the vessel for more than a few hours—sufficiently long, however, to produce a partial coagulation of blood in the sac, since the pulsation never returned.

At the site of the ligature upon the artery in Hunter's canal the coats of the vessel were deeply reddened, and it was filled with an adherent clot; yet its caliber was not diminished.

There was no trace of the cat-gut ligature. Mr. Spence used the cat-gut ligature on the common carotid. The next day the patient suddenly became comatose and paralyzed on the left side. Upon examination this artery was not in the slightest degree constricted at the point of deligation. The ligature was found with difficulty, and so gelatinous and pulpy that a portion of it, when placed between two slips of glass, spread out like a fluid. An embolic clot plugged the middle cerebral artery. The internal coats were cut through; but when the ligature softened, the force of the circulation distended the vessel, and forced the recent clot onward into a vessel of the brain.

Although Mr. Spence uses the cat-gut to tie arteries in amputation, removal of tumors, etc., he would not again use it to tie a large continuous trunk for aneurism. He now, for large arteries in amputation, uses cat-gut for the upper ligature, close to the soft parts, and dentist's silk for the lower, near the open mouth of the vessel. We fail to see any advantage in this double ligation of a vessel, especially when one of these ligatures is silk.

In the fourth case (Mr. Holden's) the patient died of hæmorrhage on the eighth day subsequent to ligation of the femoral in Scarpa's triangle, antiseptically with cat-gut. Not a trace of the ligature could be found. The hæmorrhage had taken place through a jagged perforation at the site of the ligature. The inner and middle coats were fairly divided, yet there was no clot, the vessel being pervious throughout. The cat-gut loses its constricting power, not by slipping, but by softening. With such evidence we must endorse the author's only conclusion, "that the fate or behavior of a given antiseptic cat-gut ligature, applied to the continuity of an artery, cannot be foretold."

The concluding lecture is devoted to "the ligation of a main artery to arrest acute traumatic inflammation." This operation was suggested by the author as a novelty in 1867; but he subsequently learned, and duly acknowledged the fact, that the operation originated in our own country. We have only space for the author's conclusions:

1. "That ligature of the superficial femoral artery has arrested acute inflammation consequent on wound of the knee-joint."

2. "That ligature of a main artery will generally diminish profuse suppuration, and prevent death by exhaustion."

3. "That while it arrests profuse suppuration, it will, by allowing the patient to gain strength, afford an opportunity for amputation at a future time."

4. "That gangrene and secondary hæmorrhage, as the result of ligature, should not be anticipated in the healthy subject."

5. "That a dread of these has arisen from our knowledge of the consequences of the ligature in instances of known diseased vessels."

6. "That a slough on the heel, caused by the pressure of a splint, was quickly detached, and the wound soon closed, although the superficial femoral had been tied a few days previously."

7. "That the arterial tension of the rest of the body will be increased beneficially by the ligature." J. E. O.

